# American War Nurses

**Published:** 1919-02-01

**Authors:** 


### American War Nurses.

The United States is beginning to think of some form of memorial to the nurses who have given up their lives in the service of the nation. Meanwhile, says the *Trained Nurse and Hospital Review*, "there is one thing for which we can all feel a certain responsibility—to see that no nurse who returns incapacitated for further service by the strain of war duty is neglected. The public will respond to such needs once they are made known, and once the public is awake to the importance of immediate action. It is up to every one of us to see that adequate provision is made for every returning war nurse who needs rest and opportunity to recuperate in proper surroundings. While we are getting ready to do something toward a general memorial, each school from which a nurse has died in military service may wisely plan a memorial tablet or similar device, for the nurses' home or the chapel, which will serve to bring to the nurses of the future the part played by their school in the great war."

